# Magnetic Resonance Spectrum Imaging of Endometrial Lesions: A Diagnostic Performance Comparative Study with Pathological Findings

**DOI:** 10.2174/0115734056457836260704084554

**Published:** 2026-07-14

**Authors:** Zhuoting Xu, Minhua Shen, Yue Zhou, Shuang Zhou, Guofu Zhang, He Zhang

**Affiliations:** 1 Department of Radiology, The Obstetrics and Gynecology Hospital, Fudan University, Shanghai, China; 2 Department of Gynecology, The Obstetrics and Gynecology Hospital, Fudan University, Shanghai, China; 3 Department of Radiology, Shanghai Pudong Hospital, Fudan University Affiliated Pudong Medical Center, Shanghai, China

**Keywords:** Endometrial neoplasms, Magnetic resonance imaging, Magnetic resonance spectroscopy, Biomarkers, Histopathology, Differential diagnosis

## Abstract

**Objective:**

To evaluate the diagnostic value and significance of magnetic resonance spectroscopy (MRS) in endometrial pathology.

**Methods:**

In this retrospective study, the ^1^H-MRS records of patients with suspected abnormal endometrial conditions were analyzed. Single-voxel spectra acquired from the largest region of the lesions were evaluated. The peak heights and areas under the curves (AUCs) for different metabolic peaks (total choline (tCho), creatine (Cr), lactate, lipid, N-acetylaspartate (NAA)) were recorded. The patients were classified into two groups: benign (hyperplasia or polyp) and malignant. Statistical differences in each peak between the two groups were analyzed individually.

**Results:**

Tumor lesions showed higher tCho (*p*=0.006), Cr(*p*=0.007), and lipid peaks (*p*=0.042) than benign endometrial lesions, together with increased tCho/Cr (*p*=0.021) and tCho/NAA ratios (*p*=0.016). In endometrial cancer, higher tCho(AUC=0.64), lipid (AUC=0.618), and tCho/Cr (AUC=0.649) were associated with higher tumor grade. Lipid and tCho/NAA also demonstrated value for differentiating endometrioid from non-endometrioid subtypes, with AUCs of 0.761 and 0.756, respectively. Lactate and an NAA-like peak were detectable in a subset of endometrial lesions. Multivariable logistic regression revealed that the incorporation of the proton MRS tCho metric into a baseline clinico-radiological model (age, body mass index (BMI), cancer antigen 125 (CA125)) significantly enhanced diagnostic performance (AUC = 0.797).

**Discussion:**

tCho- and lipid-related MRS metrics may aid non-invasive characterization of malignancy and tumor aggressiveness, supporting preoperative subtype assessment and risk stratification.

**Conclusion:**

MRS reflects the basic metabolic characteristics of tissues and aids in determining the nature and invasiveness of endometrial lesions.

## 1 INTRODUCTION

Endometrial cancer (EC) is the most common gynecological malignancy in developed countries, and its incidence is rising alarmingly in developing nations, primarily driven by changing dietary patterns, lifestyle shifts, and the growing prevalence of metabolic syndrome [1]. Although histopathology remains the cornerstone of diagnosis, EC management is rapidly shifting toward precision medicine. The 2023 International Federation of Gynecology and Obstetrics (FIGO) staging system now incorporates the Cancer Genome Atlas (TCGA) molecular classification, highlighting the critical prognostic differences among subtypes and the need for individualized care [2]. Standard preoperative evaluation relies on endometrial biopsy and multi-parametric Magnetic Resonance Imaging (mpMRI). However, due to the profound spatial heterogeneity of endometrial carcinomas, conventional blind biopsies risk missing aggressive clones. This frequently results in low sensitivity for focal lesions and pathological under-grading. Consequently, advanced imaging is essential to capture the macroscopic landscape of the entire tumor bed [3]. Meanwhile, the diagnostic accuracy of conventional mpMRI is frequently compromised by confounding anatomical factors, such as severe myometrial thinning and coexisting adenomyosis [4]. Preoperative differentiation of malignant from benign uterine lesions remains a key clinical objective. Diagnostic insights that help exclude malignancy could serve as a useful adjunct in determining the surgical extent, aiming to prevent unnecessary oncological overtreatment and associated morbidity in patients with benign conditions.

Driven by metabolic reprogramming, malignant transformation involves profound biochemical alterations that often precede macroscopic morphological changes detectable by conventional Magnetic resonance imaging (MRI). Acting as a non-invasive tool for *in vivo* metabolomics, Proton magnetic resonance spectroscopy (^1^H-MRS) captures these early shifts. By quantifying specific markers such as tCho for aberrant phospholipid metabolism, lipids (Lips) for lipogenesis, and creatine (Cr) ^1^H-MRS has already proven its diagnostic efficacy in neuro-oncology, breast, and prostate cancers [5]. Despite these successes, ^1^H-MRS application in uterine pathology lacks clinical standardization. Biomarkers validated in neuroglial tissues require rigorous re-evaluation in the female pelvis, where technical challenges like motion artifacts and peak overlap persist [6]. To bridge this gap, this study evaluates the diagnostic performance of *in vivo* ^1^H-MRS in differentiating malignant endometrial cancer from benign lesions. Furthermore, we investigated the utility of specific metabolic parameters including the total choline-to-creatine (tCho/Cr) ratio, total choline-to-N-acetylaspartate (tCho/NAA) ratio, and Lip peaks for stratifying tumor grades and identifying histological subtypes, offering a non-invasive adjunct for preoperative assessment.

## 2 MATERIALS AND METHODS

### 2.1 The Patients’ Selection

This single-center retrospective study evaluated a consecutive cohort of patients with suspected endometrial lesions who underwent 3.0T MRI scanning between October 2022 and August 2023. No eligible patients were arbitrarily skipped during this timeframe. In all, 67 consecutive patients with suspected endometrial tumors, after being subjected to 3.0T MRS prior to dilation and curettage, were found to be eligible. The exclusion criteria for patients included: (a) tumor diameter less than 1 cm, (b) non-endometrioid lesions, (c) incomplete pathology information, and (d) other types of malignancy, including but not limited to endometrial tumor. The patients included for analysis totaled 65.

Acquiring high-quality pelvic ^1^H-MRS data is inherently challenged by bowel gas-induced field inhomogeneity and physiological motion. Therefore, all spectra underwent rigorous quality inspection. Excluding severe artifacts was an essential quality-control step; such artifacts not only preclude accurate conventional mpMRI interpretation but also cause severe spectral baseline distortions, poor signal-to-noise ratio (SNR), and extreme peak broadening (large linewidths), rendering the data mathematically uninterpretable. Although most of the previous studies in this field have been done on smaller cohorts of subjects, *i.e*., 20–40 patients [7], in our study, we have included 65 patients, making it one of the larger cohorts in terms of numbers. This number of subjects would be statistically adequate to detect the characteristic heterogeneity of metabolism in endometrial carcinomas, making it sufficient as a basis to validate the utility of MRS in this field. A post hoc analysis revealed that our cohort of 65 patients would have >80% power to detect significant differences in metabolism.

### 2.2 Image Evaluation

The radiologist, with extensive experience, first used a three-plane approach to locate the suspicious endometrial lesions. The uterine structures were examined on axial, coronal, and sagittal views, and the cut on which the lesion was most prominently visualized was used to take the subsequent measurements. On the T2-weighted imaging (T2WI) cuts, a circular region of interest was manually placed on the lesion on the cut showing the maximum extent of the lesion, and a 2-cm diameter was used as the size of the region of interest. The maximum diameter of the lesion was manually measured on the same cut, being careful to avoid the peri-endometrial junctional zone and any fluid present within the uterine cavity. All regions of interest and lesion contours were reviewed by a fellowship-trained subspecialty radiologist (Z.H.) with over 20 years of experience. The radiologists were aware they were looking through scans performed to detect endometrial cancer but were blind to any other clinical information.

### 2.3 MRI Scan

All imaging studies were done with a 3.0T MRI machine with a 16-channel body coil system (Ingenia CX, Philips Healthcare, Netherlands). Patients were scanned in the supine position. Routine imaging studies were done in sagittal fat-suppressed T2WI, axial T1-weighted imaging (T1WI), axial fat-suppressed T2WI, and coronal T2WI. For these imaging studies, the FOV used was 250 x 250 mm with a matrix of 252 x 252 pixels, a slice thickness of 4-5 mm with an inter-slice gap of 0.8-1.0 mm. Contrast-enhanced imaging studies were done with fat-suppressed T1WI in sagittal, axial, and coronal planes. The contrast agent used was a gadolinium contrast agent (GE Healthcare, Shanghai, China), given at a rate of 2 mL/s with an injection volume of 0.2 mmol/kg body weight of the patient. FOV used was 250 x 250 mm with a matrix size of 192 x 193 pixels, a slice thickness of 3.5 mm with slice overlap for image acquisition.

^1^H-MRS was done before the application of the contrast medium. For the planning of localized spectroscopy, reference was made to the sagittal, axial, and coronal planes of the T2WI sequence. The voxel for spectroscopy was placed in the center of the tumor, avoiding adjacent non-tumor tissues as much as possible. A single-voxel sequence of Point-Resolved Spectroscopy (PRESS) was applied with a voxel size of 2.0×2.0×2.0 cm^3^, TR/TE being 2000/144 ms, and signal averages being applied at 128. Automatic shimming and water suppression were done before data acquisition. The acquisition time per voxel was 4.28 min.

The diffusion-weighted imaging (DWI) was done by means of a single-shot echo-planar imaging (EPI) technique. The DWI was done with two different b-values (50 and 1000 s/mm^2^) with TR/TE = 2980/64 ms, NEX = 4, FOV = 240 x 240 mm, and slice thickness = 5 mm. The average DWI acquisition time was approximately 1.06 min.

### 2.4 Statistical Analysis

All analyses were conducted using Python (v3.9). Data normality was assessed via the Shapiro-Wilk test. Continuous variables were compared using the independent t-test or Mann-Whitney U test, and categorical variables via the Chi-square or Fisher’s exact test, as appropriate.

To evaluate the incremental diagnostic value of ^1^H-MRS, a baseline clinical model (comprising age, body mass index (BMI), cancer antigen 125 (CA125), and tumor size) and a combined model (Baseline + tCho metric) were constructed using multivariable logistic regression (statsmodels). A total of 52 patients (36 malignant and 16 benign) with detectable total choline (tCho) peaks were included in the final diagnostic performance analysis. Diagnostic performance was quantified and compared using Receiver Operating Characteristic (ROC) curves and the Area Under the Curve (AUC). A two-tailed P < 0.05 indicated statistical significance.

## 3 RESULTS

### 3.1 Patients’ Characteristics

Clinical characteristics and tumor-related information for patients are listed in Table **1**. In total, 42 EC patients and 23 benign endometrial lesions were included for analysis. In general, more consistent results were observed for EC voxels compared to benign lesions. In EC patients, NAA and tCho peaks were observed to be detectable for most voxels, at a rate of 93% (39/42) and 86% (36/42), respectively. In contrast, Cr, lactate (Lac), and Lip peaks were observed for approximately 70-76% of voxels. For benign endometrial lesions, lower percentages of voxels for most metabolites were observed, especially for NAA and Lac. However, Lip peaks remained detectable for a relatively high percentage of benign endometrial lesions at 78% (18/23).

Voxel localization during voxel placement was performed using anatomical MR images to ensure that the voxel was positioned within the lesion while avoiding adjacent structures. Figure **1A** shows voxel placement on sagittal images in a case of complex hyperplasia, and Fig. (**1B**) shows the corresponding MRS findings. In the case of an endometrial polyp, the voxel was placed within the mass, as shown in Fig. (**2A**), with the related metabolic findings shown in Fig. (**2B**). In the case of high-grade adenocarcinoma, the voxel was placed within the solid mass, as shown in Fig. (**3A**), and the corresponding metabolic findings are shown in Fig. (**3B**).

### 3.2 MRS Metabolic Components Analysis

Univariate analysis showed that the EC group was associated with increased tCho, Cr, and Lip peaks compared with benign lesions. The AUCs for tCho, Cr, and Lip peaks were 0.730, 0.730, and 0.742, respectively, indicating moderate discriminatory performance between EC and benign lesions. The ROC curves and related diagnostic performance of these metabolic parameters are shown sequentially in Fig. (**4A** and **B**). In addition, the EC group showed an increased tCho/Cr ratio and tCho/NAA ratio, as well as a reduced NAA peak, compared with benign lesions.

In terms of tumor grade differentiation, ^1^H-MRS metrics, including tCho, Lip, and tCho/Cr, exhibited only marginal discriminatory potential, as reflected by AUC values ranging from 0.618 to 0.649 (Tables **2** and **3**; Fig. **5**).

In contrast, Lac levels did not show any significant correlation with malignancy level (*p* = 0.245, Table **2**). Of interest, although the Cr peak was higher in the EC group than in the benign lesion group, higher Cr levels within the EC group were associated with lower-grade malignancies. NAA peaks were present in both the EC and benign lesion groups, suggesting that NAA levels alone may be insufficient for distinguishing between the two groups. With AUC values of 0.761 for Lip peaks and 0.756 for tCho/NAA, these metabolic parameters showed a trend toward discriminative ability between endometrioid and non-endometrioid carcinomas, suggesting that they may serve as supplementary indicators in the differential diagnosis (Table **3**; Fig. **5**).

To determine whether ^1^H-MRS provides additional diagnostic value over conventional clinical assessment, multivariable logistic regression models were evaluated. The baseline clinical model (incorporating age, BMI, and CA125) yielded an AUC of 0.714 (Fig. **6**). When the ^1^H-MRS tCho metric was incorporated into the combined model, the diagnostic performance notably improved to an AUC of 0.797 (Fig. **6**). Notably, within the combined model, the tCho metric remained a statistically significant independent predictor of malignancy (*p* = 0.04), suggesting its potential to provide complementary diagnostic information alongside routine clinical markers.

## 4 DISCUSSION

Our findings demonstrate that EC tissues exhibit a significantly higher concentration of tCho-related compounds (including choline, glycerophosphocholine, phosphocholine, and o-phosphoethanolamine) compared to normal endometrial tissues (Table **2**). The alterations in Lip metabolism were noted across all malignant tissues and appeared to be more prominent in the higher-grade tumors (Table **3**). It is also important to note that alterations in the metabolism of tCho are not unique to EC and can be considered a generalized phenomenon of carcinogenesis, possibly a reflection of the synthetic activity of cellular membranes.

A number of studies have tried to discover biomarkers to predict progression to invasive carcinoma from hyperplasia, but most of them have not yet reached clinical utility [8]. Similar metabolic signatures have also been identified in other cancers, which indicate that these metabolic changes could be related to tumor biology in general, not specific to each cancer [9]. Early studies done by Nuclear Magnetic Resonance spectroscopy, as well as recent studies on large numbers of samples by metabolomics, have shown that phosphocholine is elevated in endometrial cancer compared to benign tissue. These studies, in total, indicate abnormal tCho metabolism as a metabolic hallmark in cancerous transformation, with several enzymes in this pathway, especially tCho kinase alpha, being potential biomarkers and targets [10]. With regard to tumor grading, in one study by Skorupa *et al*. [11], it was found that tCho was elevated in cancerous tissue compared to benign tissue, irrespective of grading. On the other hand, in this study, it was found that in grade 3, compared to other grades, there was a further increase in tCho-containing metabolites (Figs. **1B** and **3B**). The difference in findings could be due to several factors, but it is in line with recent studies on metabolomics, which have indicated that phospholipid-type metabolites could be one of the factors contributing to an aggressive phenotype in high-grade, especially in high-risk, tumors [12]. Importantly, our multivariable analysis confirmed that the tCho metric is an independent predictor of malignancy (*p* = 0.04), even after adjusting for traditional clinical confounders such as age, BMI, and CA125. This provides strong statistical evidence that quantitative ^1^H-MRS offers tangible incremental diagnostic value, reinforcing its potential as a powerful complementary tool in routine clinical workflow.

Furthermore, Cr is synthesized primarily in the liver and kidneys and subsequently transported to muscle tissues. There, it plays a central role in energy metabolism within the phosphagen system, driven by the catalytic activity of creatine kinase (CK). This system is involved in maintaining energy homeostasis within the cell. In cancer, Cr is also an energy metabolism marker that indirectly reflects information regarding cancerous tissues’ energy requirements for rapid growth [13]. Ozyurt *et al*. [14] found that endometrial polyps showed reduced peak Cr accompanied by an increase in peak Lac. These alterations may indicate changes in local energy metabolism, with reduced Cr reflecting altered phosphocreatine-dependent energy buffering and elevated Lac suggesting increased glycolytic activity and/or a relatively hypoxic and acidic microenvironment [15]. Conversely, using 3.0T MRI, we found that peak Cr was more commonly elevated in malignant EC compared to benign endometrial polyp tissues (Table **2**). These observations can be explained by postulating that benign tissues, compared to malignant tissues, have different metabolic requirements for energy production, which is necessary for continued growth and progression.

Lac, an end-product of glycolysis, reflects the Warburg effect in proliferating tumors [11, 16]. While previously reported to be elevated in EC [17]. Lac is also detectable in non-malignant endometrial processes [13]. In our study, Lac was quantified in 71% of EC and 61% of benign voxels, with the increase in EC lacking statistical significance (*p*=0.245, Table **2**). This non-significance is primarily driven by the inherent spectral overlap between the Lac doublet (1.33 ppm) and Lip methylene resonances (1.3 ppm). In conventional ^1^H-MRS, this unavoidable Lip contamination masks the true extent of tumor hypoxia. Furthermore, accurate quantification is inherently constrained by spectral quality, tissue heterogeneity, and voxel placement.

Resonances at 1.3 ppm typically reflect Lip accumulation from rapid cell turnover and necrosis [18]. Although measurable in both EC (69%) and benign controls (78%), at short echo times this region represents a lipid-dominated composite peak (Lip+Lac). Rather than a limitation, this overlap synergistically captures both necrosis (Lips) and hypoxia (Lac) hallmarks of tumor aggressiveness [19]. This biological synergy may explain its preliminary utility in differentiating highly aggressive non-endometrioid from endometrioid carcinomas (AUC = 0.761, Table **3**). However, with only four non-endometrioid cases, this finding is statistically underpowered and remains strictly exploratory, though it highlights the potential of combined metabolic profiling in EC.

In our series, Lip content in high-risk endometrial cancer was higher than that in benign endometrial lesions and appeared to increase with tumor grade (Table **3**). This finding is consistent with previous metabolomic studies [20] showing altered lipid metabolism, particularly involving phospholipids, in endometrial cancer, which may be associated with tumor progression and aggressive behavior [21]. Increased Lip signals may therefore reflect enhanced lipid metabolic reprogramming, rapid cellular turnover, membrane remodeling, and necrosis in more aggressive tumors [5].

We also observed a peak at 2.09 ppm in one individual tumor data set. Although gynecologic MRS data have historically assigned this peak to NAA [22], recent data on non-neuronal tissues challenge the validity of this assignment. Alternatively, it has been hypothesized that resonances in this range may represent N-acetylated macromolecules, such as mucins or sialic acid-containing glycoconjugates [23]. However, without *in vitro* specimen analysis or mass spectrometry validation, the exact biochemical assignment of this peak in our cohort remains uncertain and purely speculative. From a clinical perspective, this observed peak appeared in both benign and malignant data sets without correlation to tumor grade. Therefore, while it provided little utility for preoperative risk stratification in our study, future translational studies integrating *in vivo* ^1^H-MRS with ex vivo high-resolution magic angle spinning nuclear magnetic resonance and liquid chromatography-mass spectrometry on paired surgical specimens are essential to definitively resolve its precise origin.

An inverse correlation in the levels of Cr and tCho has been found in brain tumors, in which tumor tissue demonstrates increased levels of tCho with concomitant decreases in Cr compared with non-tumor tissue in the brain. Such an inverse correlation in tumor tissue has been termed a “tCho/Cr reversal” and has helped establish the rationale behind the potential value of the tCho/Cr ratio as a tumor biomarker [24]. The earlier studies had suggested particular cutoff values for spectral ratio values in tumor tissue; however, with the development and implementation of more recent MR spectroscopy techniques employing multiple parameters in tumor tissue characterization studies, it has been found that malignant tissue in ovarian tumors demonstrates increased tCho/Cr ratio values compared with benign tissue in the ovary [25]. Such increased tCho/Cr ratio values in malignant tissue have been correlated with increased membrane phospholipid metabolism in tumor tissue compared with benign tissue in the ovary, in which metabolic activity levels are significantly lower in benign tissue compared with malignant tissue in the ovary in patients with malignant ovarian tumors in our study. The tCho/Cr ratio values in benign tissue in the uterus and in malignant tissue in the endometrium in patients with endometrial cancer differed in our study, suggesting its potential value in tissue characterization in the uterus. The tCho/Cr ratio values in tumor tissue in our study correlated with tumor grade in endometrial cancer (Table **3**).

Field strength is an important factor in the reliability of quantifying metabolites in the human body using ^1^H-MRS. For us, it is advantageous to utilize a 3.0T machine as it provides us with increased SNR benefits in comparison to a 1.5T machine. This is important in the separation of resonances, particularly when trying to detect metabolites at low concentrations [14]. Although it is true that as field strength is increased, signal strength is also increased, it is important to note from previous validation studies that similar diagnostic ratios exist in 1.5T and 3.0T machines [26]. This is expected to lead to an overall reduction in the number of spectra being excluded due to quality issues.

Despite the advantages of MRS at 3.0T regarding better SNR, spectroscopic examinations of female pelvic regions are technically demanding. The anatomy of this region can sometimes cause problems regarding signal quality because of interfaces between tissues and air caused by bowel gas. In some cases, fluid collections or even metallic objects can add to problems with homogeneous B0 distribution. These problems can negatively influence spectral resolution as well as metabolite quantification accuracy. In addition to this, fat suppression can sometimes prove to be more problematic at higher fields. The strong signal obtained from fat can sometimes result in poor suppression of fat signals, leading to signal overlap from other metabolites located at similar chemical shift frequencies, such as Lac. The motion caused by bowel peristalsis and/or respiration can sometimes add to problems regarding stability during longer acquisition times [27]. These problems may partly explain our inability to quantify some metabolite signals across all cases.

## 5 STUDY LIMITATIONS

However, this study has several limitations. First, the single-center retrospective design and strict technical exclusions (*e.g*., severe motion artifacts or small lesions) introduced selection bias, limiting generalizability. Additionally, the small non-endometrioid subgroup (n=4) renders our subtype analysis strictly exploratory. Second, the single-voxel technique inherently risks sampling bias by only capturing the central tumor, thereby missing macroscopic metabolic heterogeneity. Third, metabolic interpretation is constrained by spectral overlaps (*e.g*., Lac and Lips at 1.3 ppm) and a lack of ex vivo validation for atypical resonances (*e.g*., the 2.0 ppm peak, which likely represents N-acetyl macromolecules rather than neuronal NAA). Finally, relying on qualitative visual inspections without reporting quantitative spectral metrics (*e.g*., exact SNR and linewidth) is a technical constraint. Future prospective, multicenter studies are warranted to validate these findings.

## CONCLUSION

While peak overlap and motion artifacts remain practical challenges, ^1^H-MRS offers a unique metabolic perspective that complements standard MRI. Parameters like the tCho/Cr ratio and Lip profiles can provide an additional layer of information for risk stratification, which may ultimately help refine preoperative planning particularly when fertility-sparing options are being considered.

## AUTHORS’ CONTRIBUTIONS

The authors confirm contribution to the paper as follows: H.Z., G.Z.: Study conception and design; M.S., S.Z.: Data collection; Z.X., Y.Z.: Analysis and interpretation of results; Z.X., M.S.: Draft manuscript. All authors reviewed the results and approved the final version of the manuscript.

## Figures and Tables

**Fig. (1):**
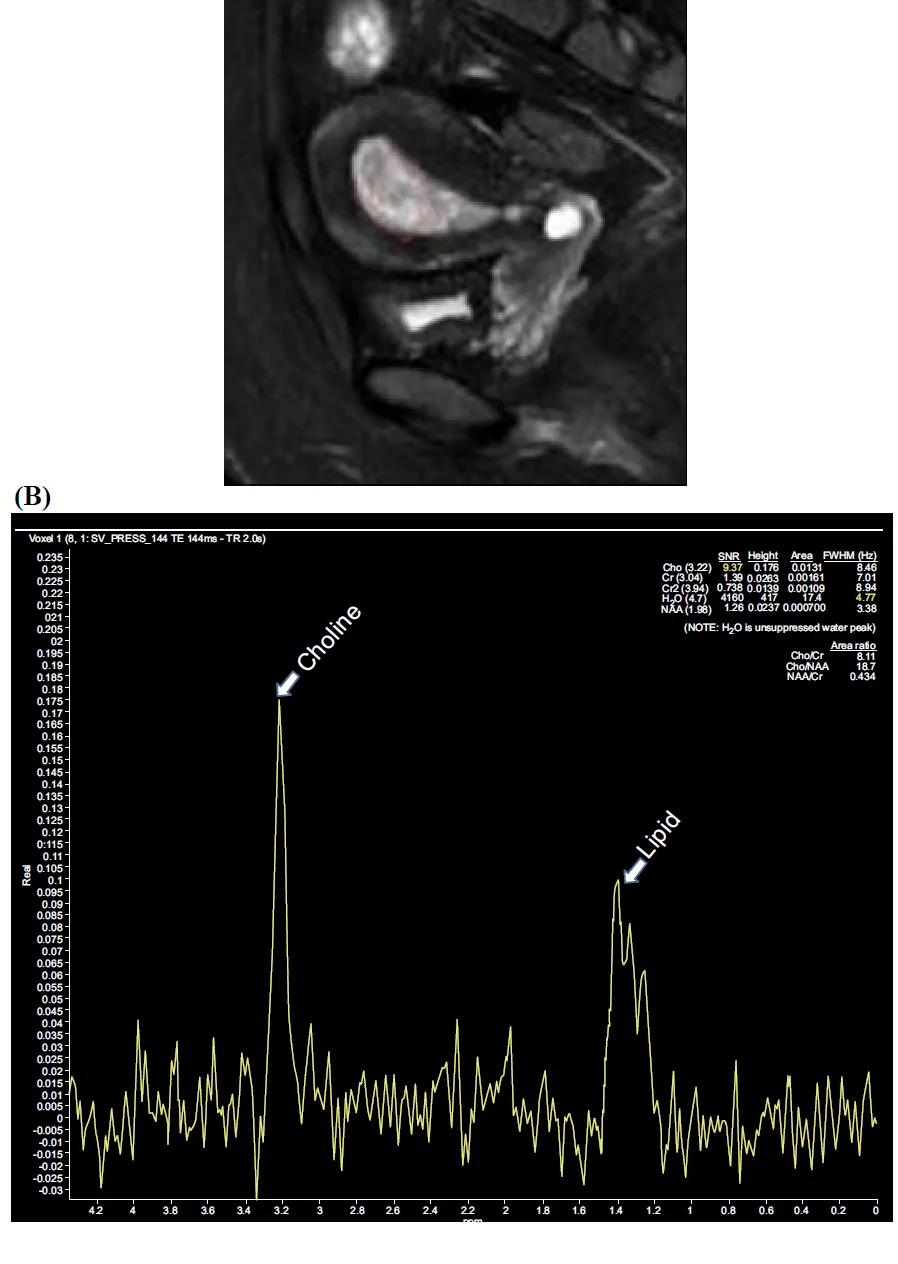
Magnetic resonance imaging and spectroscopy of complex endometrial hyperplasia. (**A**) Sagittal T2-weighted MRI demonstrating the voxel placement (spectral localization) within the endometrial lesion. (**B**) The corresponding ^1^H-MRS spectrum. The x-axis represents the chemical shift in parts per million (ppm), and the y-axis represents signal intensity. Elevated total choline (tCho) and lipid (Lip) peaks are clearly observed.

**Fig. (2):**
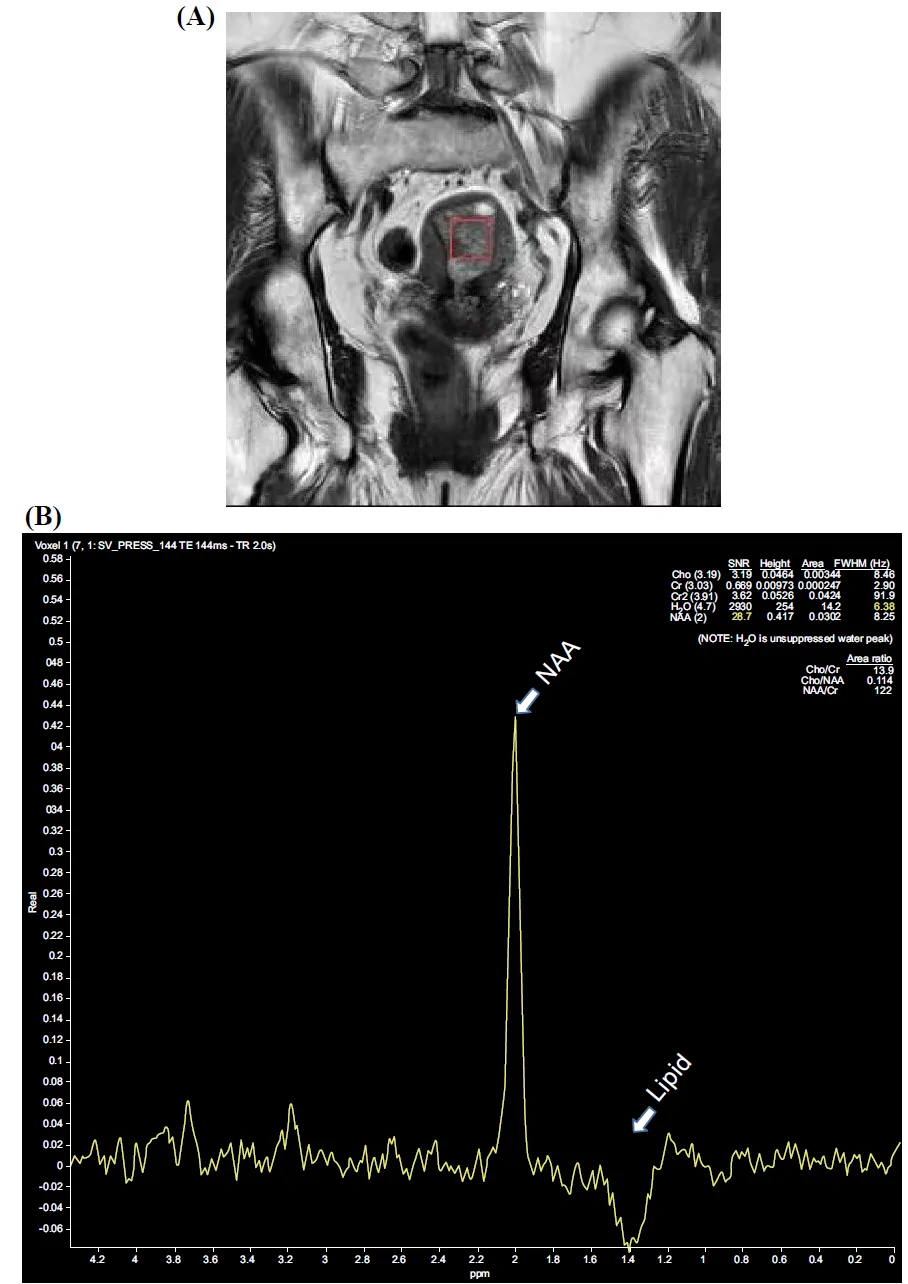
Magnetic resonance imaging and spectroscopy of an endometrial polyp. (**A**) Coronal T2-weighted MRI demonstrating voxel placement (spectral localization) within the polypoid lesion. (**B**) The corresponding ^1^H-MRS spectrum. The x-axis represents the chemical shift in parts per million (ppm), and the y-axis represents signal intensity. Elevated N-acetylaspartate (NAA) and lipid (Lip) peaks are clearly observed.

**Fig. (3):**
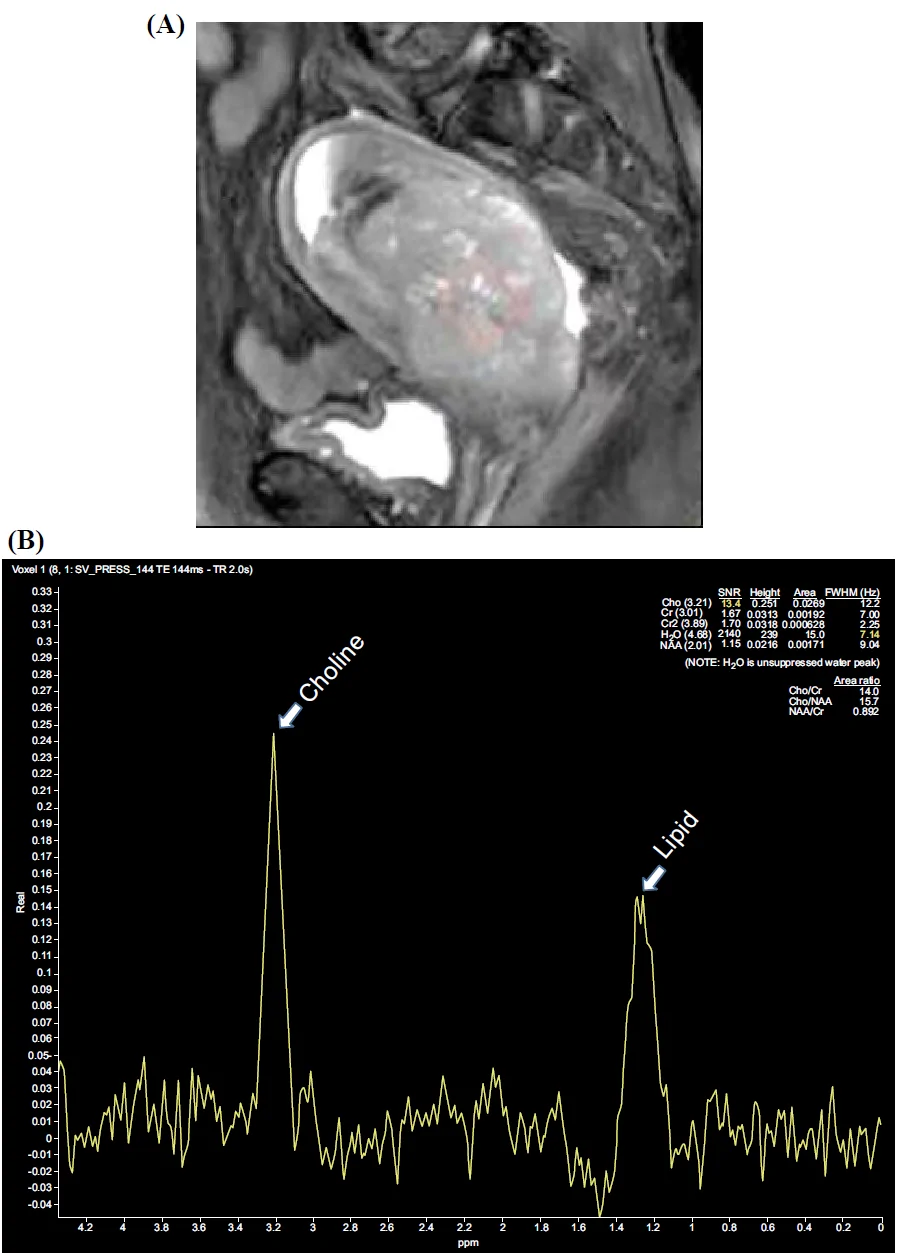
Magnetic resonance imaging and spectroscopy of a high-grade endometrial adenocarcinoma. (**A**) Sagittal MRI demonstrating the voxel placement (spectral localization) within the malignant lesion. (**B**) The corresponding ^1^H-MRS spectrum. The x-axis represents the chemical shift in parts per million (ppm), and the y-axis represents the signal intensity. Significantly elevated total choline (tCho) and Lipid (Lip) peaks are clearly observed.

**Fig. (4):**
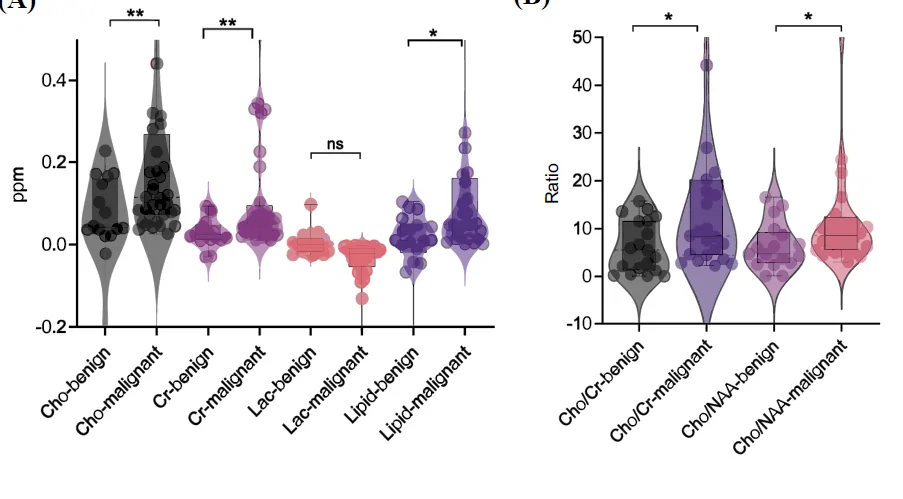
Violin plots of 1H-MRS metabolic metrics in benign and malignant endometrial lesions. The x-axis displays the evaluated metabolites or ratios stratified by group, and the y-axis represents their quantitative values in parts per million (ppm) or relative ratios. (**A**) Individual metabolite levels. Significant differences are observed between groups for tCho, Cr (** *p* < 0.01), and Lipid (* *p* < 0.05), but not for Lac (ns). (**B**) Metabolite ratios. Both tCho/NAA and tCho/Cr show statistically significant differences between benign and malignant samples. **Abbreviations:**^1^H-MRS: proton magnetic resonance spectroscopy; Cr: creatine; Lac: lactate; Lip: lipid; NAA: N-acetylaspartate; ns: not significant; tCho: total choline.

**Fig. (5):**
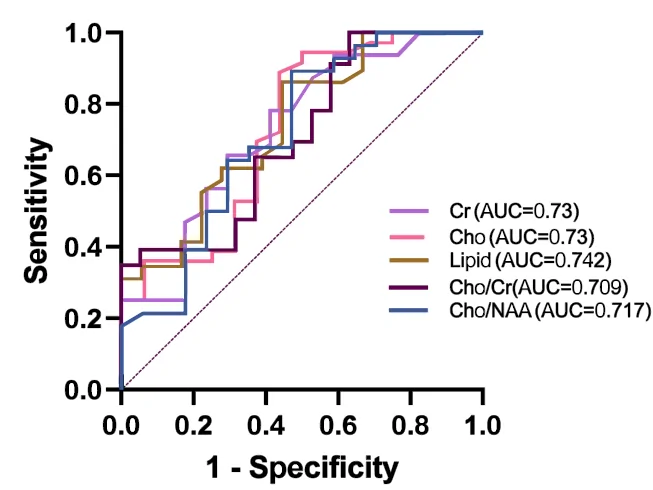
ROC curves of individual ^1^H-MRS metabolic metrics and ratios. The x-axis represents the false positive rate (1 - Specificity) and the y-axis represents the true positive rate (Sensitivity). The solid lines indicate the diagnostic performance of different parameters: light purple for Cr (AUC=0.73), pink for tCho (AUC=0.73), brown for Lip (AUC=0.742), dark purple for tCho/Cr (AUC=0.709), and dark blue for tCho/NAA (AUC=0.717). The red dashed line denotes the random reference. **Abbreviations:** ^1^H-MRS: proton magnetic resonance spectroscopy; AUC: area under the curve; Cr: creatine; Lip: lipid; NAA: N-acetylaspartate; ROC: receiver operating characteristic; tCho: total choline.

**Fig. (6):**
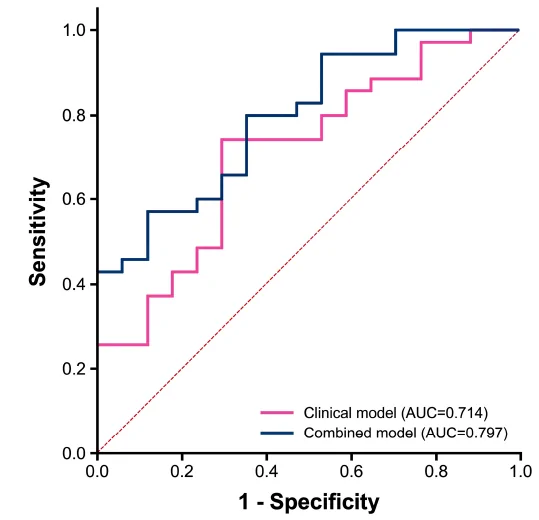
ROC curves of the clinical and combined models. The x-axis represents the false positive rate (1 - Specificity) and the y-axis represents the true positive rate (Sensitivity). The pink line indicates the baseline clinical model (age, BMI, CA125; AUC=0.714). The dark blue line represents the combined model (+ tCho metric), which shows improved performance (AUC=0.797). The red dashed line denotes the random reference. **Abbreviations:** AUC: area under the curve; BMI: body mass index; CA125: cancer antigen 125; ROC: receiver operating characteristic; tCho: total choline.

**Table 1:** Summaries of clinical characteristics for the included samples (N=65), stratified by lesion type (malignant *vs*. benign).

	**Malignant Group** (N =42^1^)	**Benign Group** (N =23^1^)	**Test value**	***P*-value^2^**
**Case, n(%)**	42 (0.65)	23 (0.35)	-	-
**Age, (year)**	51.5(37.25, 56)	38 (32,46)	-	0.042
**BMI, (kg/m^2^)**	23.55 (22.5, 27.67)	29.17 (23.5, 35.22)	-	0.083
**Ca125, (U/mL)**	21.5 (14.5, 34.25)	13.9 (10.3, 30.3)	-	0.063
**He-4, (pmol/L)**	67.4 (57.65, 100.7)	54 (47.2, 62.9)	-	0.001
**Ki-67, (%)**	40.26 ± 20.89	22.7 ± 17.4	2.44	0.019
**Histologic subtype, n(%)**	-	-	-	-
Endometrioid	38(0.9)	0	-	-
Clear cell	0 (0)	0	-	-
Serous papillary	3(0.07)	0	-	-
Others^3^	1(0.02)	0	-	-
**Histologic grade^4^, n(%)**	-	-	-	-
G1	26(0.68)	0	-	-
G2	2(0.05)	0	-	-
G3	9(0.24)	0	-	-
**FIGO stage^4^, n(%)**	-	-	-	-
I	32(0.78)	0	-	-
II	2(0.05)	0	-	-
III	7(0.17)	0	-	-
IV	0	0	-	-

**Note:** ^1^Data presented as mean ± SD for continuous variables and n (%) for categorical variables. ^2^P values: Student's t-test for continuous variables, Fisher's exact test for Hemorrhage and Menopausal Status, chi-square test for other categorical variables. ^3^others: Carcinosarcoma. ^4^The total number of cases for histologic grade (n=37) and FIGO stage (n=41) differ due to missing data in some pathological records and because non-endometrioid carcinomas are not routinely graded.

**Table 2:** The quantitative parameters measurements on *in vivo* MRS in the two subgroups.

	**Malignant Group(N=42^1^)**	**Benign Group(N=23^1^)**	**test value^1^**	***P*-value^2^**
**Creatine**	-	-	-	-
Peak(cm^3^)	0.056 (0.027,0.19)	0.026 (0.014,0.064)	-	0.007*
Area	0.004 (0.002,0.017)	0.003 (0.001,0.007)	-	0.019*
**Choline**	-	-	-	-
Peak	0.218±0.192	0.084±0.132	-2.84	0.006*
Area	0.014 (0.008,0.026)	0.011 (0.003,0.015)	-	0.035*
**Lipid**	-	-	-	-
Peak	0.198±0.416	0.009±0.085	-2.088	0.042*
Area	0.013 (0.008,0.03)	0.007 (0.004,0.027)	-	0.034*
**Lactic acid**	-	-	-	-
Peak	-0.019±0.276	-0.009±0.037	1.175	0.245
Area	0.004 (0.001,0.01)	0.007 (0.003,0.04)	-	0.029*
**Cho/Cr**	-	-	-	-
Ratio	8.530 (4.585,19.100)	5.580 (1.515,11.600)	-	0.021*
**Cho/NAA**	-	-	-	-
Ratio	8.560 (5.555,11.450)	4.720(2.840,8.740)	-	0.016*

**Note:** ^1^Data are presented as mean ± standard deviation or median (interquartile range), as appropriate. Group comparisons were made using the Independent Samples t-test or the Mann-Whitney U test. ^2^P values: Student's t-test for continuous variables, Fisher's exact test for Hemorrhage and Menopausal Status, chi-square test for other categorical variables. *: indicates a statistically significant difference.

**Table 3:** Diagnostic performance of MRS parameters in determining the endometrical malignancies in the included sample (N =65).

**Group**	**Creatine**	**Choline**	**Lipid**	**Cho/Cr**	**Cho/NAA**
**Malignant /Benign**	-	-	-	-	-
ROC^1^	0.73	0.73	0.742	0.709	0.717
ACC^2^	0.714	0.789	0.745	0.691	0.733
**Grade1-2/3**	-	-	-	-	-
ROC^1^	0.431	0.64*	0.618*	0.649*	0.556
ACC^2^	0.769	0.75	0.763	0.794	0.745
**Endometrioid/Others**	-	-	-	-	-
ROC^1^	0.303	0.398	0.761*	0.423	0.756*
ACC^2^	0.863	0.615	0.76	0.261	0.511

**Note:** ^1^ROC:A graphical plot that illustrates the diagnostic ability of a binary classifier system by plotting the true positive rate against the false positive rate at various threshold settings. ^2^ACC:The proportion of true results (both true positives and true negatives) among the total number of cases examined. *: indicates a statistically significant difference.

## Data Availability

The authors declare that all data supporting the findings of this study are available within the article.

## LIST OF ABBREVIATIONS

AUC: Area under the curve
BMI: Body mass index
CA125: Cancer antigen 125
CK: Creatine kinase
Cr: Creatine
DWI: Diffusion-weighted imaging
EC: Endometrial cancer
FIGO: International Federation of Gynecology and Obstetrics
^1^H-MRS: Proton magnetic resonance spectroscopy
Lac: Lactate
Lips: Lipid
mpMRI: Multiparametric magnetic resonance imaging
MRS: Magnetic resonance spectroscopy
NAA: N-acetylaspartate
PRESS: Point-Resolved Spectroscopy
ROC: Receiver operating characteristic
SNR: Signal-to-noise ratio
tCho: Total choline
T1WI: T1-weighted imaging
T2WI: T2-weighted imaging
TCGA: The Cancer Genome Atlas

## STANDARDS OF REPORTING

STROBE guidelines were followed.
